# Genetic Association Analysis Reveals Differences in the Contribution of *NOD2* Variants to the Clinical Phenotypes of Orofacial Granulomatosis

**DOI:** 10.1097/MIB.0000000000000844

**Published:** 2016-06-10

**Authors:** Alexander Mentzer, Shalini Nayee, Yasmin Omar, Esther Hullah, Kirstin Taylor, Rishi Goel, Hannah Bye, Tarik Shembesh, Timothy R. Elliott, Helen Campbell, Pritash Patel, Anita Nolan, John Mansfield, Stephen Challacombe, Michael Escudier, Christopher G. Mathew, Jeremy D. Sanderson, Natalie J. Prescott

**Affiliations:** * Department of Gastroenterology, Guy's and St. Thomas' NHS Foundation Trust and King's College London, London, United Kingdom;; † Department of Medical and Molecular Genetics, Faculty of Life Science and Medicine, King's College London, Guy's Hospital, London, United Kingdom;; ‡ Oral Medicine, Guy's and St. Thomas' NHS Foundation Trust and King's College London, London, United Kingdom;; § Diabetes and Nutritional Sciences Division, King's College London, London, United Kingdom;; ‖ Faculty of Health and Environmental Sciences, AUT University, Auckland, New Zealand;; ¶ Institute of Genetic Medicine, Newcastle University, Newcastle Upon Tyne, United Kingdom; and; ** Sydney Brenner Institute for Molecular Bioscience, Faculty of Health Sciences, University of Witwatersrand, South Africa.

**Keywords:** orofacial granulomatosis, Crohn's disease, NOD2 gene, genetic association

## Abstract

**Background:**

Orofacial granulomatosis (OFG) is a rare, inflammatory disorder of the mouth, in which some patients also have intestinal Crohn's disease (CD). The etiology remains largely unknown, although there is a high prevalence of atopy, and oral granulomas are also seen in other immune disorders particularly CD and sarcoidosis. We investigated whether genetic variants associated with an increased risk of CD, sarcoidosis, or atopy were also associated with susceptibility to OFG.

**Methods:**

Patients were stratified clinically as isolated oral manifestations (OFG only) or concurrent intestinal CD (OFG+CD). We genotyped 201 patients and 1023 healthy controls for risk variants in *NOD2, IRGM, IL23R, ATG16L1* (CD), *BTNL2* (sarcoidosis), and *FLG* (atopy). The coding regions of the *NOD2* gene were screened for rare, potentially pathogenic variants in OFG.

**Results:**

A combined analysis of 3 CD-risk variants in *NOD2* showed no association with any OFG subgroup. *NOD2* p.L1007insC was associated with OFG+CD (*P* = 0.023) and *IL23R* p.R381Q with all OFG (*P* = 0.031). The sarcoidosis risk variant rs2076530 in *BTNL2* was associated with all OFG (*P* = 0.013). We identified 7 rare missense *NOD2* alleles in 8 individuals with OFG, 4 OFG-only patients and 4 patients with OFG+CD. There was a significant enrichment of *NOD2* variants in the OFG+CD group compared to the OFG-only group (*P* = 0.008, common variants; *P* = 0.04, all common and rare variants).

**Conclusions:**

Our findings suggest that genetic variants in *NOD2* are only associated with OFG in patients with concurrent intestinal disease. A genome-wide association scan is needed to fully define the genetic architecture of OFG.

## Abstract 2

Supplemental Digital Content is Available in the Text.

Article first published online 10 June 2016.

Orofacial granulomatosis (OFG) is a chronic inflammatory disorder affecting the mouth and perioral region, typically presenting with swelling of the lips and inflammation of the gingivae, buccal mucosa, floor of mouth, and other sites within the oral cavity.^1^ The precise incidence and prevalence of OFG are unknown, although it is rarely seen in general medical or dental practice and is managed by hospital specialists in secondary and tertiary care environments. Guy's Hospital in London is a specialist center for OFG and has provided care for more than 250 patients from throughout the United Kingdom.

The term OFG has been used classically to describe oral pathology in the absence of any other systemic illness, but the disease may occur in a manner clinically and histologically indistinguishable from Crohn's disease (CD) of the mouth. Indeed, some centers now classify the disease into 3 categories: OFG alone, OFG with intestinal CD, and OFG with gastrointestinal granulomata but no symptoms of intestinal CD.^2,3^ Approximately 10% of adult and 20% of child patients may progress from having OFG alone to the development of intestinal CD and patients with CD may also develop OFG, but it is very difficult to predict the course of disease in any particular patient.^4^

Other findings also present compelling reasons to question whether OFG is simply a localized disorder.^2,5^ Ileocolonoscopy performed on 35 OFG-only individuals revealed that 19 had asymptomatic macroscopic and microscopic intestinal abnormalities.^2^ This raises the possibility that OFG is a manifestation of an alternate form of CD, with an attenuated intestinal phenotype but a more severe oral phenotype. However, intestinal biopsies from these 19 patients also showed histological features that differ from the characteristic features of CD, leading to the proposal that OFG could represent a novel form of inflammatory bowel disease.^2^ A recent study examining the clinical characteristics of 21 OFG patients with concomitant CD from Sweden showed that, when compared with those with CD alone, this group represented a distinctive subphenotype of CD, particularly with respect to increased perianal disease, ileocolonic inflammation, and intestinal granulomata.^6^

The etiology of OFG is unknown. There has been no formal study investigating the heritability of OFG, and it does not seem to segregate in a Mendelian manner. Although it is now assumed that OFG is a complex trait, there have been few attempts to identify genetic susceptibility variants. It has been suggested that certain HLA serotypes may have a role in the etiology of OFG,^7^ but these findings have not been replicated to date. Unlike OFG, the genetic contribution to CD pathogenesis has been much more widely investigated. The first and most extensively replicated genetic findings in CD have been the association of 3 loss-of-function variants in *NOD2* (nucleotide oligomerisation domain containing gene 2; p.R702W, p.G908R, and p.L1007insC), a gene important in mediating the early antimicrobial response.^8–11^ Cumulatively, these variants represent the strongest genetic risk factors identified for CD,^12^ and the many genome-wide association studies performed to date have yet to find a locus with a higher attributable risk. Further DNA sequencing studies have identified another 6 missense and presumably loss-of-function variants in *NOD2* that are also significantly associated with increased CD risk,^13,14^ increasing the count of known causal variants in this gene to at least 9.

The close clinical association between CD and OFG suggests that they may have a similar genetic predisposition. Interestingly, a recent study of 29 OFG cases in Sweden investigated the role of the 3 major known CD-risk variants in *NOD2* by analysis of each variant (p.R702W, p.G908R, and p.L1007insC). They found that 4 of 12 OFG patients with concurrent CD carried one of these variants, whereas none of the 17 OFG patients without concurrent CD carried any risk variant, suggesting that these 2 groups of patients might be genetically distinct.^15^ However, the more recently discovered CD-associated *NOD2* variants (p.M863V, p.D825S, p.R703C, p.S431L, p.V793M, and p.R311W) were not considered in this study; neither was the possible contribution of the novel variants in *NOD2*. The authors emphasized that these findings needed to be confirmed in a larger cohort of well-defined patients.

Genome-wide studies have been very successful in revealing new important molecular pathways thought to contribute to the pathogenesis of CD. The association of SNPs at the interleukin-23 receptor gene (*IL23R*) locus with CD^16^ fits with the emerging major role of IL-23 and Th17 cells in mucosal inflammation.^17^ In addition, associations of coding or regulatory variants within *ATG16L1*^18,19^ and *IRGM*^20,21^ identified the link between CD and bacterial clearance.^22,23^ A recent meta-analysis of genome-wide association studies on CD by the International IBD Genetics Consortium, followed by extensive confirmation of association signals in more than 75,000 individuals has increased the number of CD-associated loci to 140^24^ and observed that many of these loci are also implicated in other immune-mediated disorders.

In addition to the overlap in clinical phenotype between OFG and CD, oral granulomata may also be found in a variety of other disorders, including *Mycobacterium tuberculosis* infection, Melkersson–Rosenthal syndrome, cheilitis granulomatosa, and sarcoidosis. Sarcoidosis is a complex multisystemic immune disorder with a reported genetic etiology^25^ that predominantly affects not only the lung but also the face and skin. One of the most well-replicated genetic associations in adult sarcoidosis involves an exonic splice site variant in exon 5 of the *BTNL2* (butyrophilin-like protein 2) gene, which results in loss of function due to truncation of the protein product,^26,27^ although the association may reflect linkage disequilibrium with the nearby HLA-DRB1.^28^ Butyrophilins are implicated in T-cell inhibition and the modulation of epithelial cell–T-cell interactions,^29^ and we have shown recently that a rare variant in *BTNL2* is associated with inflammatory bowel disease.^30^

A further component of the pathogenesis of OFG is the increased prevalence of allergy with a high incidence of concurrent hay fever, asthma, atopic dermatitis, elevated serum immunoglobulin E (Ig E), and dietary allergies.^4,31,32^ This suggests that OFG has an allergy-mediated pathogenesis, perhaps as a delayed hypersensitivity reaction.^31^ This possibility is supported further by the discovery of a novel class of dendritic B lymphocytes that preferentially express IgE in OFG biopsy specimens.^33^ One form of atopy that has been demonstrated to have a strong genetic predisposition is atopic dermatitis. Loss-of-function mutations in the *FLG* (filaggrin) gene, including p.R501X and c.2282del4, are important risk factors in the etiology of sporadic and familial forms of atopic dermatitis and asthma.^34,35^ Filaggrin is a filament-aggregating protein, important in the formation of the cornified cell envelope and has a key role in maintaining barrier function.

The histological similarity between the granulomatous lesions in OFG, CD, and sarcoidosis, the close clinical association between OFG and CD, and the frequent association with atopy seen in OFG led us to the hypothesis that these disorders may have genetic risk factors in common. We therefore tested genetic variants known to be strongly associated with an increased risk of CD (*NOD2, IRGM, IL23R,* and *ATG16L1*), sarcoidosis (*BTNL2*), and atopy (*FLG*) for association with OFG. Also, in view of the known contribution of rare genetic variants in the *NOD2* gene to the development of CD, we screened all coding regions of *NOD2* for additional rare, known, or novel genetic variants in OFG or the combined phenotype of CD and OFG (CD+OFG).

## METHODS

### Patient Selection and Phenotype Determination

Two hundred one patients with a diagnosis of OFG with and without symptoms of intestinal CD were recruited into the study from joint oral medicine/gastroenterology clinics at Guy's and St. Thomas Hospitals, London, and The Newcastle upon Tyne Hospitals after informed consent in accordance with the local ethics committees (REC Number: 12/YH/0172). Oral medicine specialists and gastroenterologists experienced in the management of OFG and CD classified each patient into one of 2 phenotypic categories based on clinical features and specific investigations. The phenotypic categories included OFG only (OFG without any intestinal symptoms, n = 111) and OFG+CD (oral manifestations consistent with a diagnosis of OFG with endoscopic and/or radiological evidence of intestinal CD, n = 90). The presence of microscopic granulomata alone in otherwise normal intestinal mucosa, with no intestinal symptoms, was considered insufficient for a classification of OFG+CD in this study. DNA from 1023 population controls was obtained from the 1958 British Birth Cohort, which includes subjects born in 1 week of March 1958 in England, Scotland, and Wales as previously described.^36^

### Genetic Analysis

Genomic DNA was extracted from 10 mL of whole blood and genotyped for risk variants strongly associated with CD (rs2066847*/NOD2* p.Leu1007fs, rs2066845/*NOD2* p.G908R, rs2066844/*NOD2* p.R702W, rs11209026/*IL23R* p.R381Q, rs13361189/*IRGM* upstream, and rs2241880/*ATG16L1* p.T300A), sarcoidosis (rs2076530/*BTNL2* c.1078A>G), or atopic dermatitis (*FLG* p.R501X and c.2282del4) using Applied Biosystems TaqMan assays (Life Technologies). All variants tested were in Hardy–Weinberg equilibrium in cases and controls (Hardy–Weinberg equilibrium *P* > 0.05) and had a greater than 95% genotype pass rate for each variant.

The 12 exons of *NOD2* were amplified by PCR in 17 amplicons as described previously^37^ in 201 patients with OFG. These were then subjected to Sanger sequencing using a ABI Big Dye v3.0 and ABI 3730XL DNA analyzer.

### Statistical Analysis

Single-marker allele frequencies in the risk genes of interest were compared using χ^2^ statistics on SNPs with a minor allele frequency of >1% and with Hardy–Weinberg equilibrium *P* > 0.05. Genes containing multiple coding variants (*NOD2* and *FLG*) were analyzed in a composite (gene-burden) manner to assess whether the total number of risk alleles for that gene was higher in cases compared with controls. All comparisons were performed for all OFG cases (n = 201) and the 2 subphenotypes of OFG only (n = 111) and OFG+CD (n = 90). All *P* values and odds ratios (ORs) were calculated using the R program (http://www.r-project.org/). Based on published ORs for each disease-specific variant, we had >99% power to detect an association at *NOD2* (for the 3 CD variants combined), 98% for *BTNL2*, 86% for *IL23R*, and 78% for *FLG* 2282del. The power to detect association at the remaining loci is shown in Table, Supplemental Digital Content 1, http://links.lww.com/IBD/B286, which contains the results of all power calculations for all tested loci.

### Pathogenicity Prediction

A combination of 9 online pathogenicity prediction software programs were used (SIFT, PROVEAN, PANTHER, PhD-SNP, SNAP, MetaSNP, MAPP, PolyPhen1, and PolyPhen2) to predict the potential impact of each *NOD2* missense variant on gene function. These were run either independently or in parallel by means of the online consensus SNP/variant analyzer tools MetaSNP (http://snps.biofold.org/meta-snp/) and PredictSNP (http://loschmidt.chemi.muni.cz/predictsnp/). Variants were considered pathogenic if the overall consensus of both Meta-SNP and PredictSNP was “Disease”/“Deleterious.”^38,39^

## RESULTS

DNA samples from 201 patients (111 OFG only and 90 OFG+CD) were genotyped for variants known to be strongly associated with an increased risk of CD (*NOD2, IRGM, IL23R,* and *ATG16L1*), sarcoidosis (*BTNL2*), and atopy (*FLG*). A comparison of the case–control allele frequencies for each SNP is provided in Table 1. We observed suggestive association of the CD-risk alleles at *NOD2* p.L1007insC and *IL23R* p.R381Q with OFG in the presence of concurrent CD (OFG+CD; *P* = 0.023 and *P* = 0.038, respectively). In addition, we observed association of the *IL23R* p.R381Q risk variant when all 201 OFG cases were considered regardless of intestinal phenotype (*P* = 0.031), but no association was detected for the OFG-only group (*P* = 0.32). Because all 3 of the common *NOD2* CD variants that were genotyped are independently associated with CD, we also tested the combined *NOD2* genotype (any of the 3 risk alleles) for association, but none was observed for either of the OFG subphenotypes or for all OFG.

**TABLE 1.**
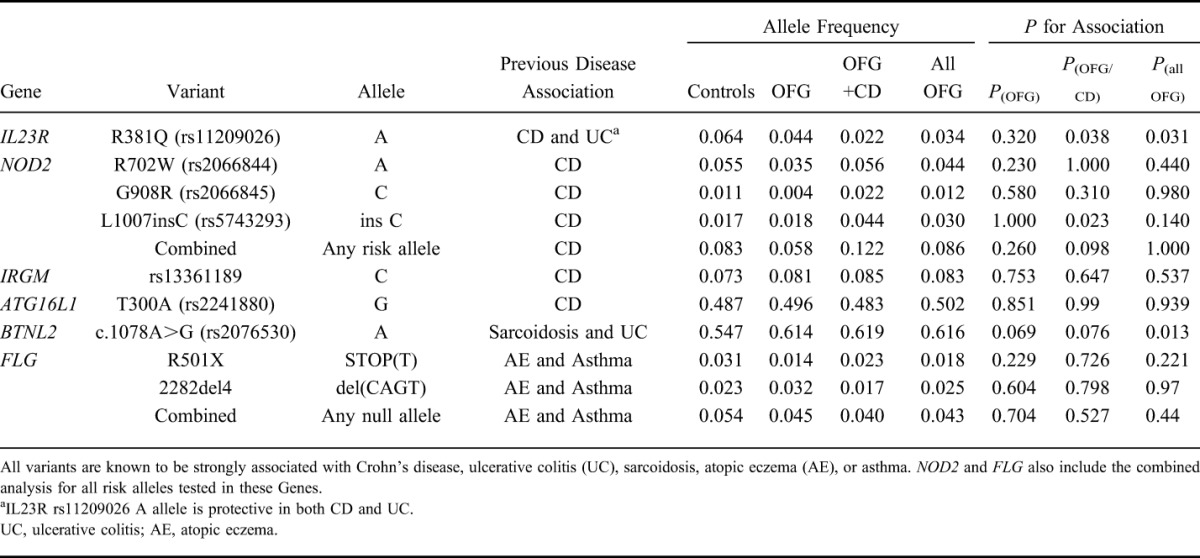
Association Analysis of 9 Risk Variants from 6 Candidate Genes in 201 OFG Cases, i.e., 90 OFG with Crohn's Disease (OFG+CD), 111 OFG Only, and 1023 Population Controls

Sequencing of all the coding regions of the *NOD2* gene identified 15 missense variants (Table 2), 10 of which were rare (≤0.1%) and occurred in only 1 or 2 individuals. A total of 7 rare variants were considered to be potentially pathogenic because they were predicted to be damaging/disease causing by the consensus of the online pathogenicity prediction tools (Materials and Methods); these were p.A181G, p.S431L, p.A616E, p.V793M, p.G908C, p.A976T, and p.L1018F. These included 2 rare variants previously identified as associated with risk of CD (p.S431L and p.V793M^14^), 1 rare variant, p.G908C, which results in a different amino acid substitution at a previously known CD-causing variant (p.G908R), and 1 novel variant p.A616E. Excluding p.S431L and p.V793M, the remaining 5 potentially pathogenic variants have not previously been implicated in CD etiology nor reported in any of the large sequencing studies of *NOD2* that have been performed in large CD cohorts.^9,13,14,37^ The novel variant, p.A616E, identified in an OFG-only individual has no entry in the Exome Aggregation Consortium database^40^—which contains exome sequencing data from >60,000 individuals—and is located in the NACHT domain of *NOD2*. The 4 other rare deleterious variants were identified in one OFG-only individual (p.G908C) and 4 individuals with OFG+CD (p.A181G, p.A976T, and p.L1018F). These 4 have been reported in the Exome Aggregation Consortium database^40^ but at extremely low frequency; p.A181G = 0.003%, p.G908C = 0.03%, p.A976T = 0.02%, and p.L1018F = 0.003% compared with our observed frequency in OFG which was 10- to 100-fold greater (Table 2). Three of these variants (p.G908C, p.A976T, and p.L1018F) are in the leucine-rich repeat domain of *NOD2*, which also harbors the 3 common CD-risk variants (p.R702W, p.G908R, and p.L1007insC), whereas the other 2 are located in the CARD2 domain (p.A181G) and NACHT domain (p.A616E).

**TABLE 2.**
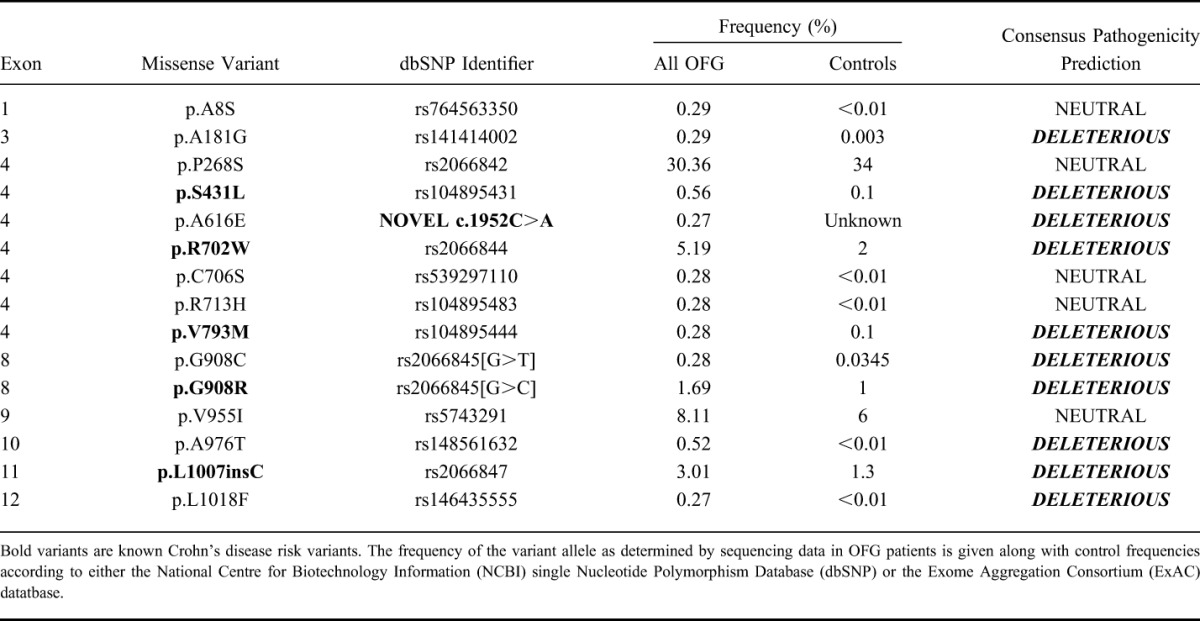
All *NOD2* Variants Detected by Sanger Sequencing of 201 OFG Cases

We observed all 3 of the common CD-risk variants in both OFG subgroups: p.R702W (OFG only = 6/111; OFG+CD = 9/90), p.G908R (OFG only = 1/111; OFG+CD = 4/90), and p.L1007insC (OFG only = 4/111; OFG+CD = 8/90) (Table 3). Overall, there was a significant difference in the cumulative frequency of *NOD2* variation (*P* = 0.008) at these 3 loci between OFG-only patients (11/111, 9.9%) and patients with OFG+CD (21/90, 23.3%). If we also consider the 7 rare and potentially pathogenic *NOD2* variants, then these cumulative frequencies increase to 13.5% (15/111) of the OFG-only patients and 27.7% (25/90) of the patients with OFG+CD, but the difference remains significant (*P* = 0.009). The remaining 2 common missense variants detected by *NOD2* sequencing in OFG were known polymorphisms and considered by pathogenicity prediction tools to be benign or tolerated and have no functional consequences (p.P268S and p.V955I).

**TABLE 3.**
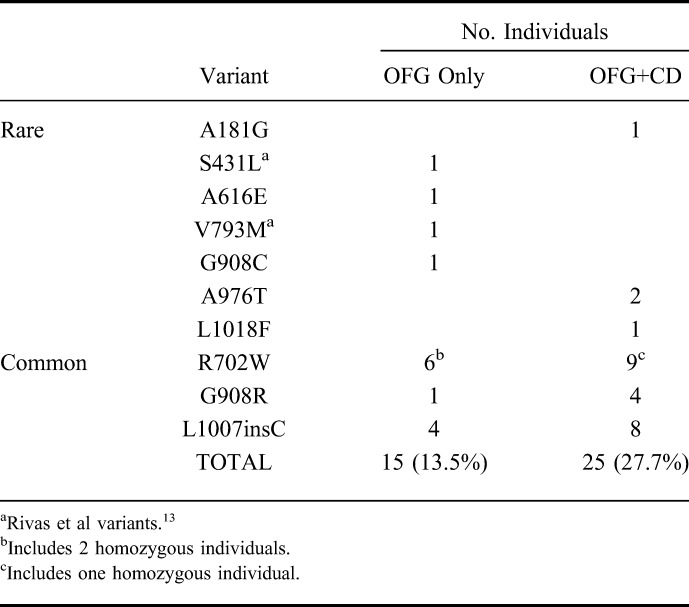
The Number of Occurrences of Each of the Rare and Common Pathogenic *NOD2* Missense Variants in OFG-Only Patients (OFG, n = 111) or OFG Patients with Concurrent Crohn's Disease (OFG+CD, n = 90)

The sarcoidosis risk variant *BTNL2* c.1078G>A (rs2076530) was associated with disease in all OFG cases (*P* = 0.013; OR = 1.33; 95% CI, 1.06–1.67) and had a similar OR (1.56) and the same direction of effect as seen in sarcoidosis.^27^ However, no association was seen for the subphenotypes of OFG only or OFG+CD. There was no association of the atopy risk variants in the *FLG* gene with OFG for the individual variants or for the combined analysis of both variants.

## DISCUSSION

The aim of this study was to begin to address the paucity in knowledge of the potential genetic basis of OFG. We analyzed a panel of genetic markers known to be associated with an increased risk of clinical phenotypes, which overlap with OFG. Although this is a rare disorder, our case–control study had reasonable power to detect association for most variants tested based on their reported effect sizes in CD, sarcoidosis, and atopy, especially if they were reasonably common and/or had a higher risk for disease. Power varied from 67% to 100% at the defined alpha-level (α = 0.05) in the whole OFG group, except for the CD-risk variant at *IRGM* (40%) and the AD risk variant p.R501X at *FLG* gene (43%). However, power in the smaller OFG-only group was lower, with a range of 30%–99% for all variants tested. Genetic effect sizes in common complex disorders are generally low and will therefore be very difficult to detect in a rare disease, such as OFG. However, it is possible that the variants that predispose to this condition are themselves rare, with larger effects sizes, which would make the detection of such effects in smaller sample sizes achievable.

The combined analysis of the 3 common *NOD2* CD-risk variants showed no association with either OFG alone, OFG+CD, or all OFG, despite being well powered to do so (power > 99%). This suggests that *NOD2* does not make a substantial contribution to OFG risk. However, there was evidence of association of the p.L1007insC variant in the OFG+CD group, and the association showed a similar OR of 2.67 (95% CI, 1.22–5.85) and direction of effect to CD. This was also the case for *IL23R* p.R381Q, which was associated in the OFG+CD group with an OR of 0.34 (0.12–0.92), showing a similar protective effect to that seen in CD. The other 2 CD-risk loci examined, *ATG16L1* and *IRGM*, did not show any association with subgroups or with all OFG although this could have been a result of the reduced power at these loci. Thus, evidence of association of CD-risk variants with disease in this study was restricted to subgroups that included patients with intestinal inflammation (CD+OFG or all OFG). This finding could be explained by insufficient power to detect association in the OFG-only group (discussed above) or that OFG only is a genetically distinguishable disease entity, a view that is consistent with our previous clinically based observations (Campbell et al, 2011).

The other positive finding in our study was the association of the variant rs2076530 in *BTNL2* with OFG in our full panel of OFG cases but not in either of the subphenotype categories (with or without intestinal CD). This variant has been associated with susceptibility to adult-onset sarcoidosis in multiple studies.^26,27^ Sarcoidosis and CD have been found to occur together in families.^41,42^ This led to the suggestion of an overlapping genetic etiology for these 2 disorders, which is supported by our results. The risk allele causes formation of a truncated gene product which displays abnormal cellular localization.^27^

The BTNL2 protein is proposed to function as a costimulatory molecule for T cells under physiological conditions. *BTNL2* negatively regulates T-cell activation independently of CD28 and CTLA-4 is predominantly expressed in gastrointestinal tissues and is overexpressed in mouse models of colitis.^43^ Recently, it has been shown that *BTNL2* promotes the expression of Foxp3, a transcription factor required for regulatory T-cell development and function.^44^ Thus, mutations in *BTNL2* may affect its ability to regulate T-cell activation in response to mucosal inflammation. We recently reported the association of a rare variant in *BTNL2* with CD and ulcerative colitis.^30^ The rare missense variant (p.G454C) is not in linkage disequilibrium with the common splicing variant, and is thus likely to be an independent effect, but further supports a potential role for *BTNL2* in these related disorders. However, *BTNL2* is located on chromosome 6p21 very close to the human MHC class II region. This complex region of the genome exhibits extensive linkage disequilibrium, and therefore it is important to establish independence of the association of the *BTNL2* risk allele from nearby MHC class II genes. This will require additional fine mapping studies that target this region.

An important question arising from the genetic data is whether the lack of association of the 3 common *NOD2* risk variants in the OFG-only group (Table 1) implies a real difference in the underlying pathogenesis of OFG and CD. Although the sample size of OFG-only cases was small (N = 111), we had high power to detect the large effect sizes for *NOD2* risk variants in this subgroup (>90%), which suggests either that *NOD2* does not contribute to the development of OFG or that the effect sizes are much smaller for these variants than in CD. An alternative analysis to address this question was used by Gale et al,^15^ who compared the proportion of cases in the OFG-only group with that in the CD+OFG group that carried any of the 3 common NOD2 risk variants, found that although 4 of 12 patients with OFG+CD carried one of these variants, they were absent in all 17 OFG-only patients. We compared 111 patients with OFG with 90 patients with OFG+CD and found a significantly lower frequency of common *NOD2* mutations in the OFG-only group versus OFG+CD (10% versus 23%, *P* = 0.008), which is broadly consistent with the data of Gale et al. However, our *NOD2* sequencing data, which identified 3 rare and likely pathogenic *NOD2* variants in 4 of 90 cases in the OFG+CD cohort (p.A181G, p.A976T, and p.L1018F) and 4 rare and likely pathogenic *NOD2* variants in 4 of 111 OFG-only patients (p.S431L, p.A616E, p.V793M, and p.G908C), suggest that rare *NOD2* variants might play a role in OFG only. Nevertheless, inclusion of all 7 rare and potentially pathogenic variants along with the 3 common CD-risk variants in a combined analysis of all *NOD2* risk alleles results in a lower cumulative risk allele frequency of 7.7% in the OFG-only group compared with 14.4% in the OFG+CD group (*P* = 0.04). This suggests that *NOD2* may be an important factor in distinguishing OFG+CD from OFG only.

It has repeatedly been reported that patients with CD and *NOD2* variants are more likely to have the ileal form of the disease and develop fibrostenosis requiring surgical resection,^45,46^ but any association with oral involvement has not been previously described. There is evidence that the *NOD2* p.L1007insC variant associated with OFG+CD in this study, in addition to the 2 other major CD-risk variants occurring in *NOD2*, results in a failure of the appropriate primary immune response to bacterial components such as muramyl dipeptide, and attenuated activation of the *ATG16L1* autophagy pathway,^47^ or alternatively may result in a gain-of-function downregulating the normally inhibitory cytokine, Il-10.^48^ The true mechanism remains unclear, but nevertheless, the end result is chronic inflammation of the intestinal tract in individuals with CD. Our results suggest that missense coding variation in *NOD2* in patients with OFG may reflect the presence of intestinal disease.

The observation that *NOD2* is associated with the development of OFG+CD but not OFG only in this study supports the possibility that these disorders may be genetically distinguishable once a systematic investigation of genetic risk factors in OFG has been performed. The finding of substantial differences in the genetic architecture of OFG could have significant diagnostic and therapeutic implications because patients with OFG may be best treated with therapy different from that used for CD. For example, there is good evidence that patients with OFG respond to diets excluding cinnamon and benzoates,^3,49^ whereas the presence of CD in conjunction with OFG predicts a response to a typical CD therapy.^50,51^

Overall, these observations regarding the genetics of OFG suggest that OFG and CD are relatively distinct disease entities. Nonetheless, a genetic contribution to the pathogenesis of OFG remains highly plausible as is the case for most chronic inflammatory conditions. A logical next step in the work presented here would be to expand the cohort of well-characterized patients with OFG to the point where a well-powered genome-wide association study could be performed to better define the genetics of OFG.

## Supplementary Material

SUPPLEMENTARY MATERIAL
